# Michigan State Board of Health

**Published:** 1881-08

**Authors:** Henry B. Baker

**Affiliations:** Secretary; Lansing


### Society Reports.

---

ARTICLE IX.

LANSING, June 13, 1881.

MEMBERS OF THE MICHIGAN STATE BOARD OF HEALTH,

*Gentlemen:*—At a meeting of the New Orleans Medical and Surgical Association, held May 7, the following resolution was adopted:

"*Resolved,* That the special attention of the National Board of Health be called to the unusual prevalence, at the present time, of typhus fever, scarlet fever, small-pox, and other contagious diseases in the Northern and in some of the Western States. We, therefore, urge the necessity of immediate action, the most rigid railroad inspection from such places, believing, as we do, that they may be considered *dangerously infected.*"

There have been reported from various portions of this State, cases of small-pox, so that now it may be considered to have been pretty well scattered over the State. So far, the local boards of health have succeeded in keeping the disease from spreading to an alarming extent. In this they have been aided by the fact that by action of this Board in years past, thousands of people in the State were vaccinated, and thousands more have been vaccinated this spring. But, although no general outbreak of the disease has occurred, yet, from the great numbers of immigrants pouring into and through this State, and the general prevalence of small-pox in foreign ports from whence these immigrants come, there is danger of such an outbreak, and it seems to me some action should be taken to prevent the further introduction of small-pox into the State. In order that there may be careful thought on this subject before action, I send you the copy of the following letter from Stephen Smith, M.D., Committee of the National Board of Health on the Prevention of Small-Pox, and would respectfully suggest that you give this plan some thought, and be prepared to take some action at the regular meeting of the Board, July 12, 1881. The letter is as follows :

"*Dear Dr. Baker* :—The only plan by which small-pox, spread by emigrants, can be brought under effectual control, seems to me to be this, viz., the concerted action of municipal, State, and National sanitary authorities. The special functions of each might thus be stated: 1. The National Board might appoint Port Inspectors who should examine every immigrant, and give cards to each with name and date of inspection ; if the person is found protected, give him a white card ; if unprotected, vaccinate him and give him a red card with name and date of vaccination. 2. Appoint Inland Inspectors, to be located at the great centers where the emigrant trains pass from one State into another, as at Buffalo, Ogdensburg, Pittsburgh, Wheeling, Detroit, Cincinnati, Chicago, St. Paul, Kansas City, St. Louis. These inspectors could examine the cards, allow those holding white ones to pass, but re-examine those holding red ones, and if vaccination is not effectual, re-vaccinate. State authorities could inspect at such points as the emigrants are likely to be diverted from the train and locate in the State. 3. The municipal authorities would follow the emigrant to his home and secure general vaccination of the community.

"Would your State Board be interested in taking action in this matter, or, do you not suffer from immigrants? The National Board cannot properly act unless State and local boards call for aid. If there was combined action of the State Boards, the National Board could coöperate at ports and on inter-State lines, and the whole machinery of sanitary organization could work in harmony and effectually exterminate this pestilence. Emigrants arrive at New York at the rate of 6,000 and 7,000 daily—large numbers unvaccinated, and all have been exposed to small-pox in foreign parts. It seems to me that there is a grand opportunity for the sanitary authorities of the United States to combine, each working in its sphere, and give an example of their power to exterminate a plague which will increase for a year or more unless we make such an effort. I have detailed this plan to our Health Officer and Dr. Harris, and they approve. I think our State Board will ask the National Board to aid in preventing the introduction of small-pox.

(Signed) "STEPHEN SMITH."

Such an inspection service as detailed need not confine its work to small-pox, but the introduction of typhus fever and other contagious diseases could be guarded against.

I trust this important subject will receive due attention.

Very respectfully,

HENRY B. BAKER, *Secretary.*

